# Supplementary material for: The Mechanism of Action of SAAP-148 Antimicrobial Peptide as Studied with NMR and Molecular Dynamics Simulations
Source: Pharmaceutics. 2023 Feb 24;15(3):761. doi: 10.3390/pharmaceutics15030761 (PMC10051583; doi:10.3390/pharmaceutics15030761)
Supplement: Supplementary file 1 [file pharmaceutics-15-00761-s001.zip › pharmaceutics-2203804-supplementary.pdf]

Table S1. Chemical shift of NH, Hα and Cα obtained experimentally (Spectra), for a random coil structure (POTENCI) or for an alpha-helix (ShiftX).

**Amino-acid**		**Spectra**			**POTENCI**			**ShiftX**		
Name	Num	NH	Hα	Cα	NH	Hα	Cα	NH	Hα	Cα
L	1	8.79	4.10	57.9	-	-	-	8.18	4.33	57.4
K	2	8.74	4.06	59.9	8.27	4.31	56.2	8.50	4.13	58.7
R	3	7.81	4.14	58.7	8.14	4.24	56.1	7.75	4.05	59.1
V	4	7.85	3.83	65.9	7.96	4.01	62.3	8.13	4.45	65.6
W	5	8.36	4.53	59.4	8.01	4.63	57.2	8.59	4.25	60.7
K	6	7.93	3.80	61.5	7.90	4.17	56.2	8.19	4.07	59.2
R	7	7.49	4.14	58.7	7.99	4.23	56.1	7.84	3.74	59.7
V	8	8.29	3.59	66.5	7.97	4.03	62.2	8.08	4.38	66.3
F	9	8.77	4.01	60.6	8.14	4.59	57.6	8.12	4.03	61.1
K	10	7.59	3.78	60.0	8.01	4.22	56.1	8.08	3.96	59.8
L	11	7.56	4.10	57.9	8.03	4.26	55.3	7.74	4.25	57.8
L	12	8.45	4.10	57.9	8.03	4.29	55.3	8.15	4.25	57.9
K	13	8.22	3.87	60.1	8.04	4.23	56.3	8.32	4.15	59.9
R	14	7.63	4.14	58.7	8.06	4.17	56.1	8.15	4.00	59.3
Y	15	8.11	4.39	61.0	7.97	4.46	57.9	7.78	4.34	61.2
W	16	8.71	4.36	60.2	7.79	4.59	57.1	8.06	4.44	61.0
R	17	7.95	3.85	58.6	7.84	4.14	56.2	8.19	4.15	59.6
Q	18	7.70	4.15	57.0	8.08	4.22	56.0	7.93	4.12	58.3
L	19	7.58	4.10	55.8	8.12	4.29	55.3	7.97	4.14	58.0
K	20	7.59	4.04	59.1	8.13	4.27	56.0	8.21	4.02	59.3
K	21	7.81	4.45	54.7	8.14	4.56	54.1	7.94	4.12	60.2
P	22	-	4.47	63.0	0.00	4.40	63.1	-	4.41	66.0
V	23	8.15	4.10	62.3	8.17	4.09	62.2	8.10	3.94	65.7
R	24	8.29	4.45	54.7	8.32	4.34	55.7	7.86	4.16	55.7

- : Non determined.

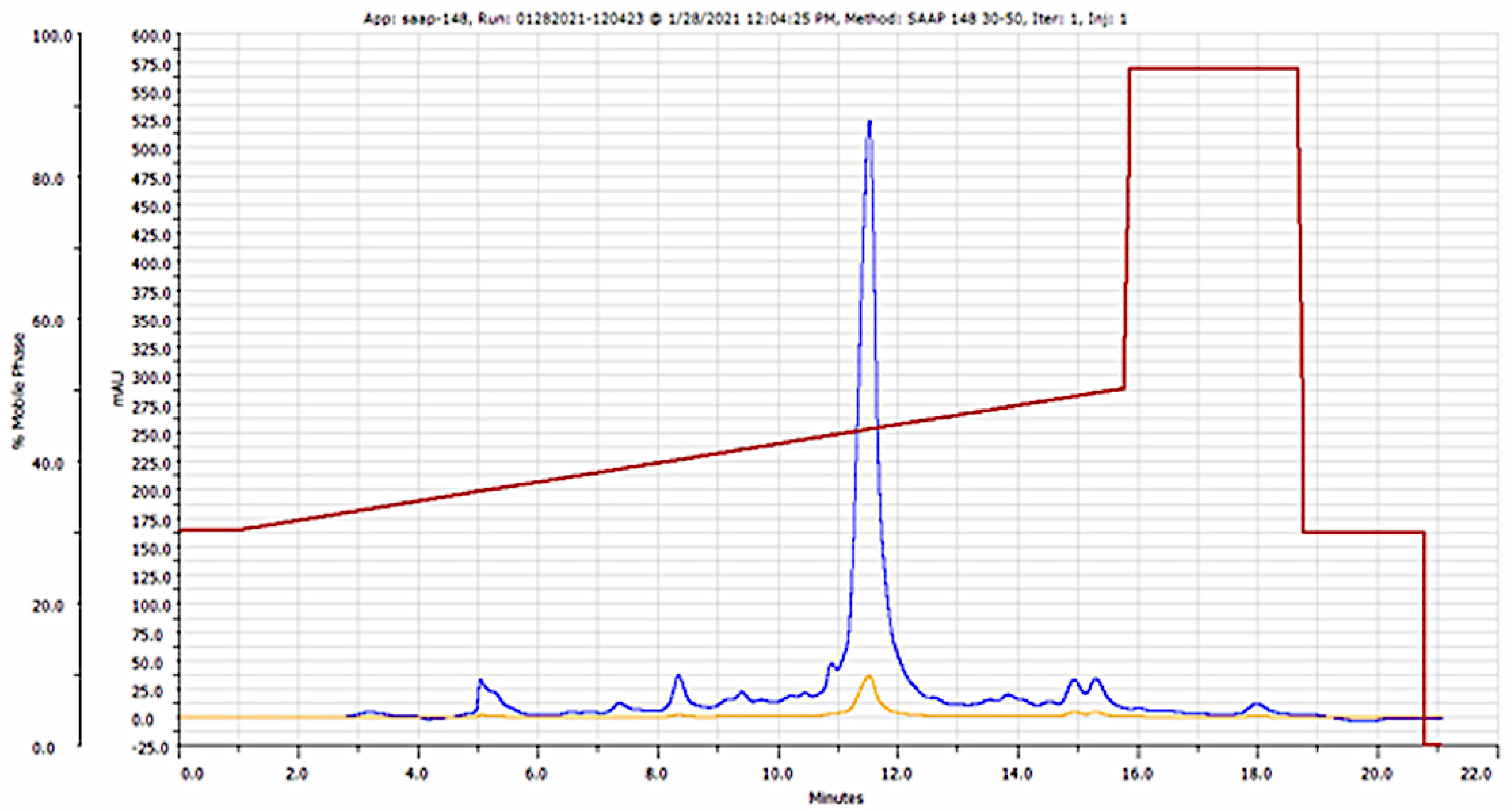

Figure S1. Analytical purity of SAAP-148 peptide: HPLC chromatogram. The peptide was purified by reverse phase HPLC (Gilson, Villiers-le-bel, France) using a preparative C18 column (Luna, C-18-100 Å-5 mm, Phenomenex, Le Pecq, France) and an acetonitrile/water gradient.

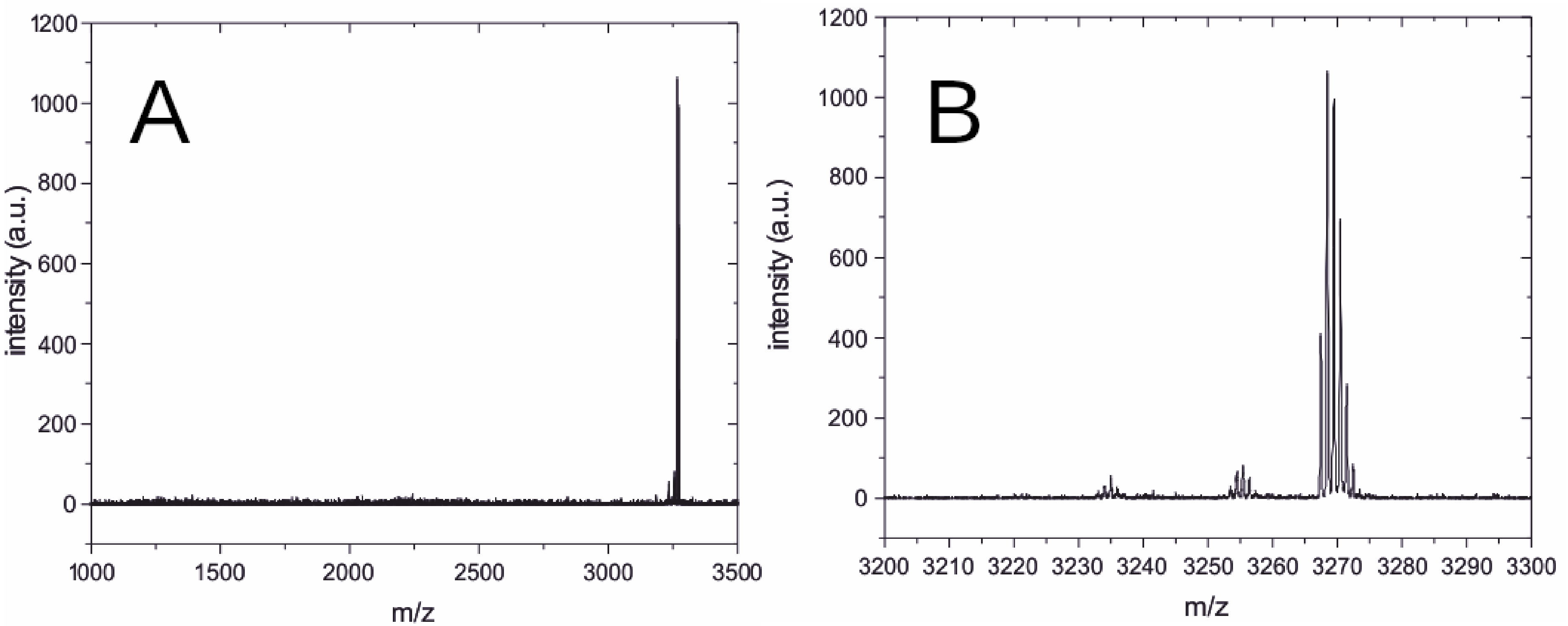

Figure S2. Analytical purity of SAAP-148 peptide: MALDI-TOF mass (A) with zoom (B) in the relevant region.

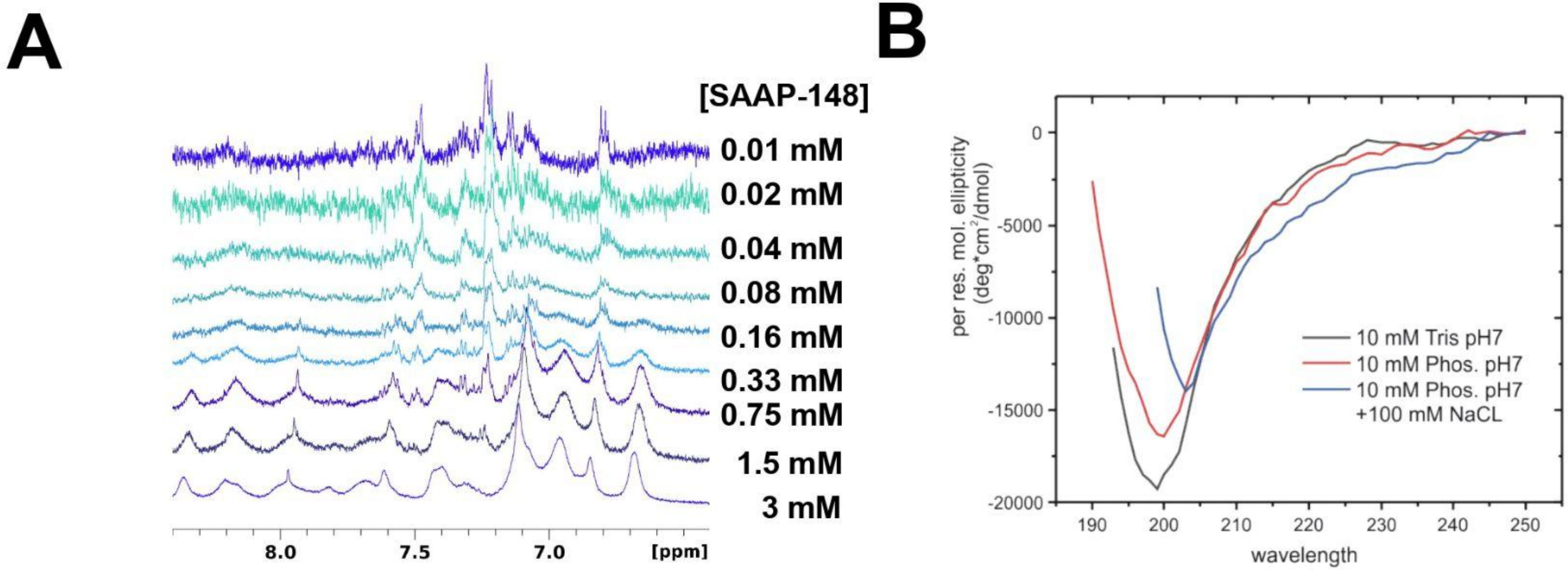

Figure S3. (A) Amino/aromatic region of 500 MHz ^1H spectrum of SAAP-148 in phosphate buffer 50 mM at pH 6.6 (H_2O/10%D_2O) at 310K as a function of concentration. (B) Normalized circular dichroism spectra of 50μM SAAP-148 in 10 mM Tris pH7 (black), 10mM phosphate buffer pH7 (red) or 10mM phosphate buffer pH7 + 100mM NaCl (blue). The temperature was 310K.

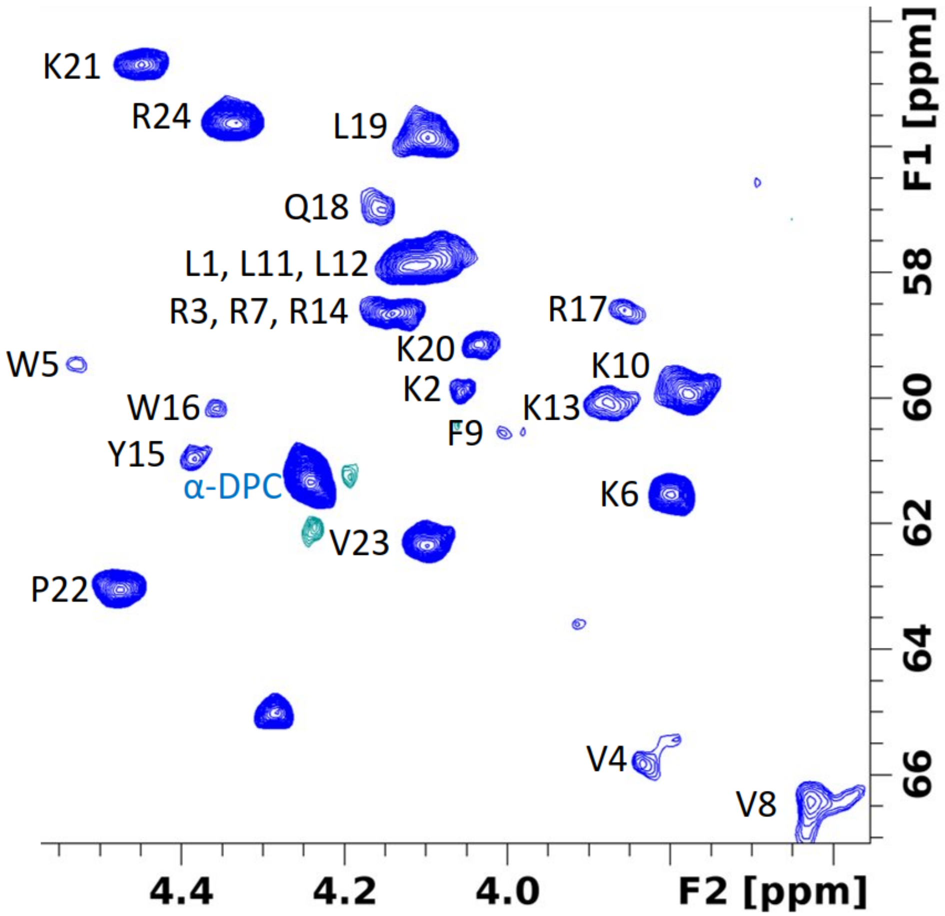

Figure S4. Hα/Cα region of 500 MHz ^1H,^{13}C-HSQC spectrum of SAAP-148 1.3 mM in DPC-d38 micelles. The assignment of non-glycine residues (whose signal is outside the chosen region) is shown on each peak.

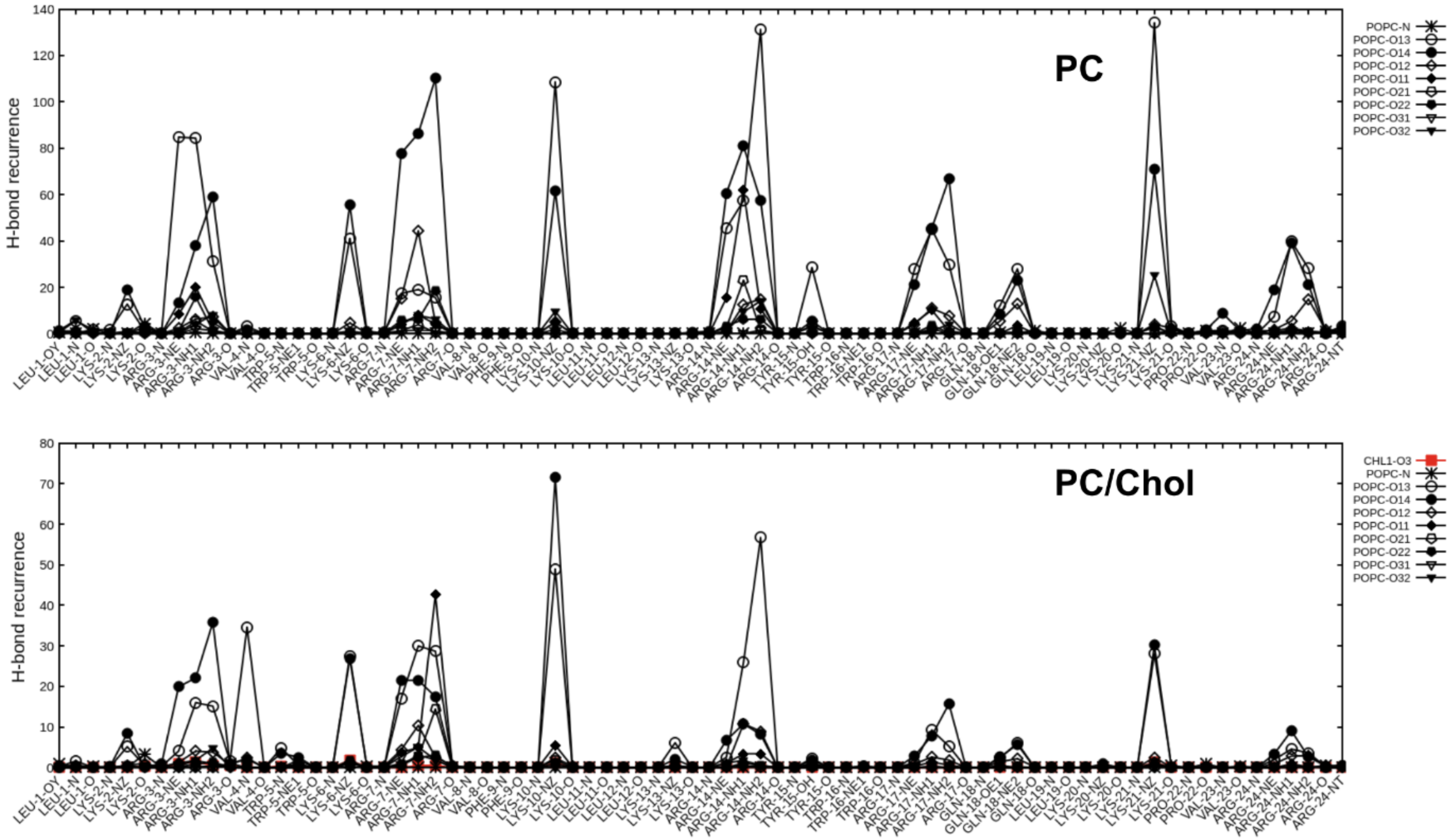

Figure S5. Polar contacts (hydrogen bonds and salt bridges) of SAAP-148 with different lipid bilayers. Sodium was used as a counterion.

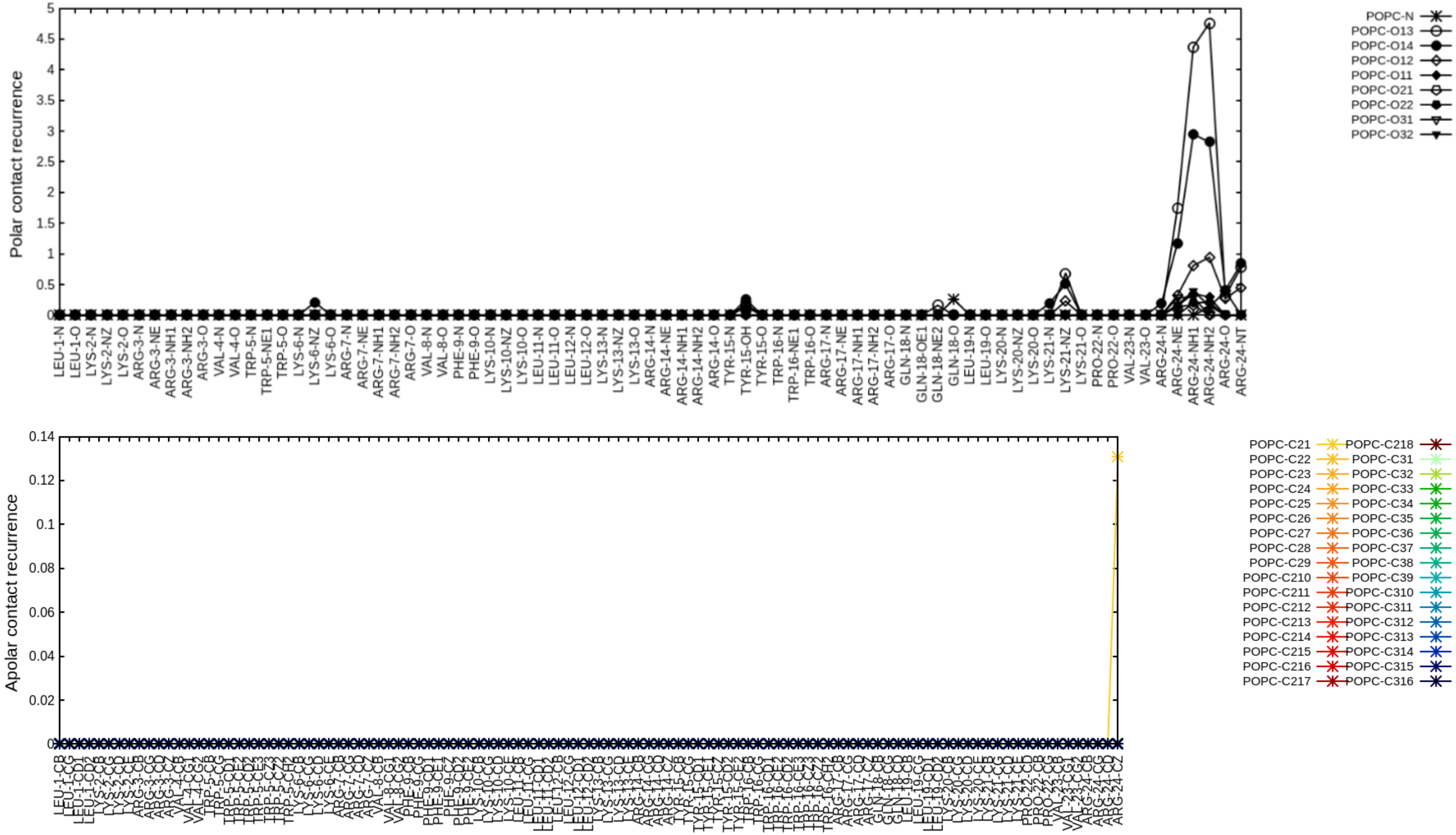

Figure S6. Polar (hydrogen bonds and salt bridges) and apolar (van der Waals interactions) contacts of SAAP-148 with POPC in presence of calcium.

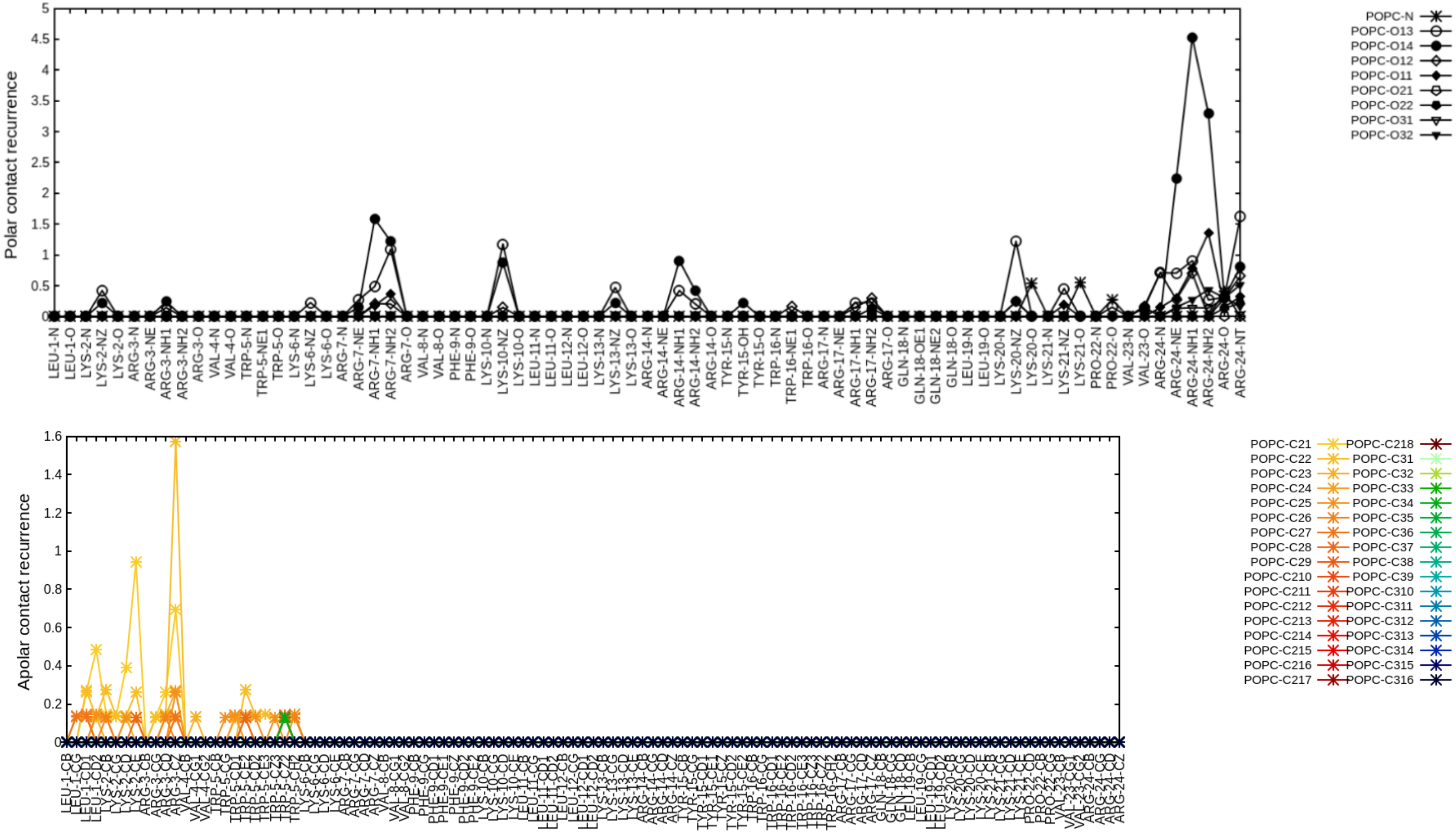

Figure S7. Polar (hydrogen bonds and salt bridges) and apolar (van der Waals interactions) contacts of SAAP-148 with POPC in presence of magnesium.

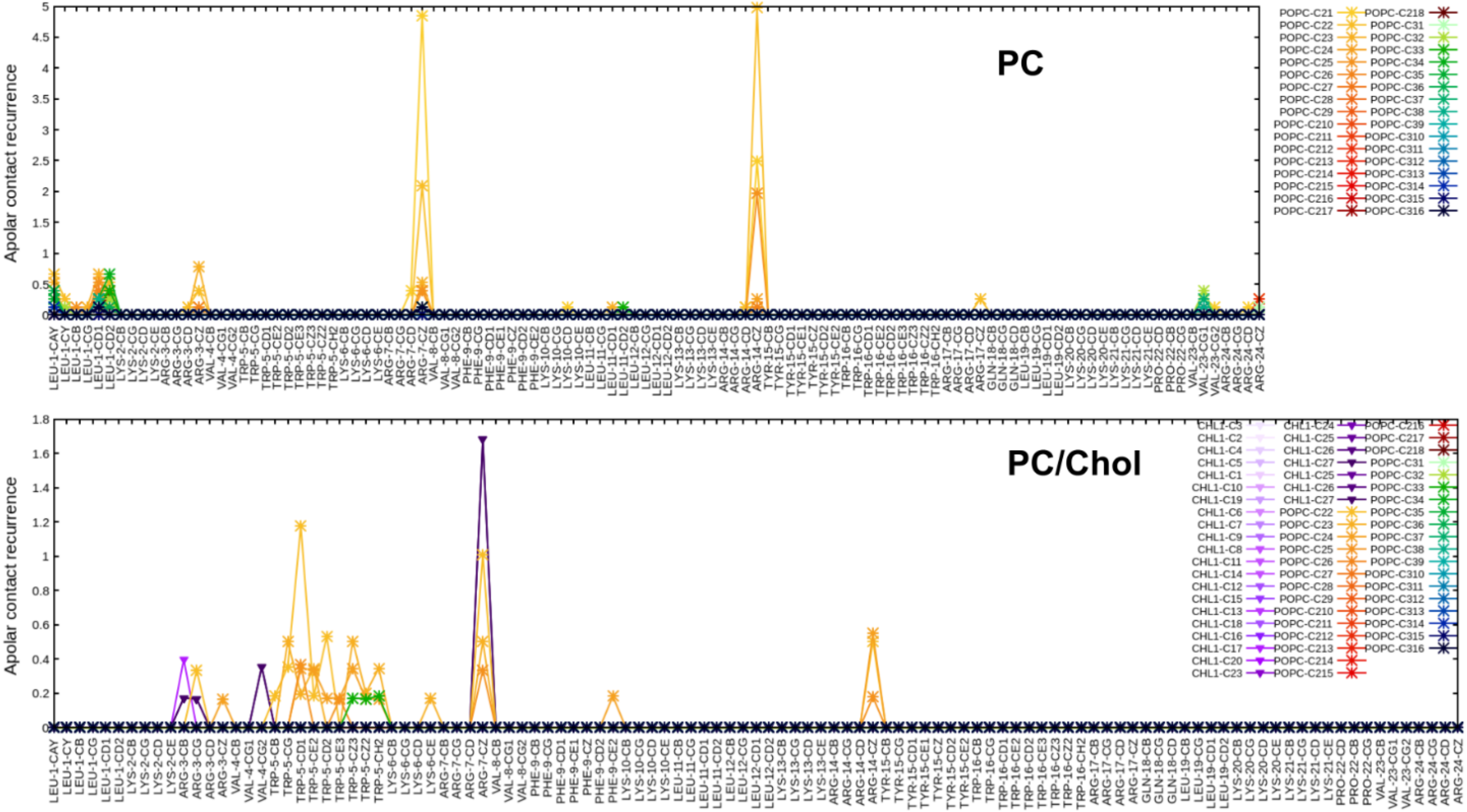

Figure S8. Apolar contacts (van der Waals interactions) of SAAP-148 with different lipid bilayers. Sodium was used as a counterion.

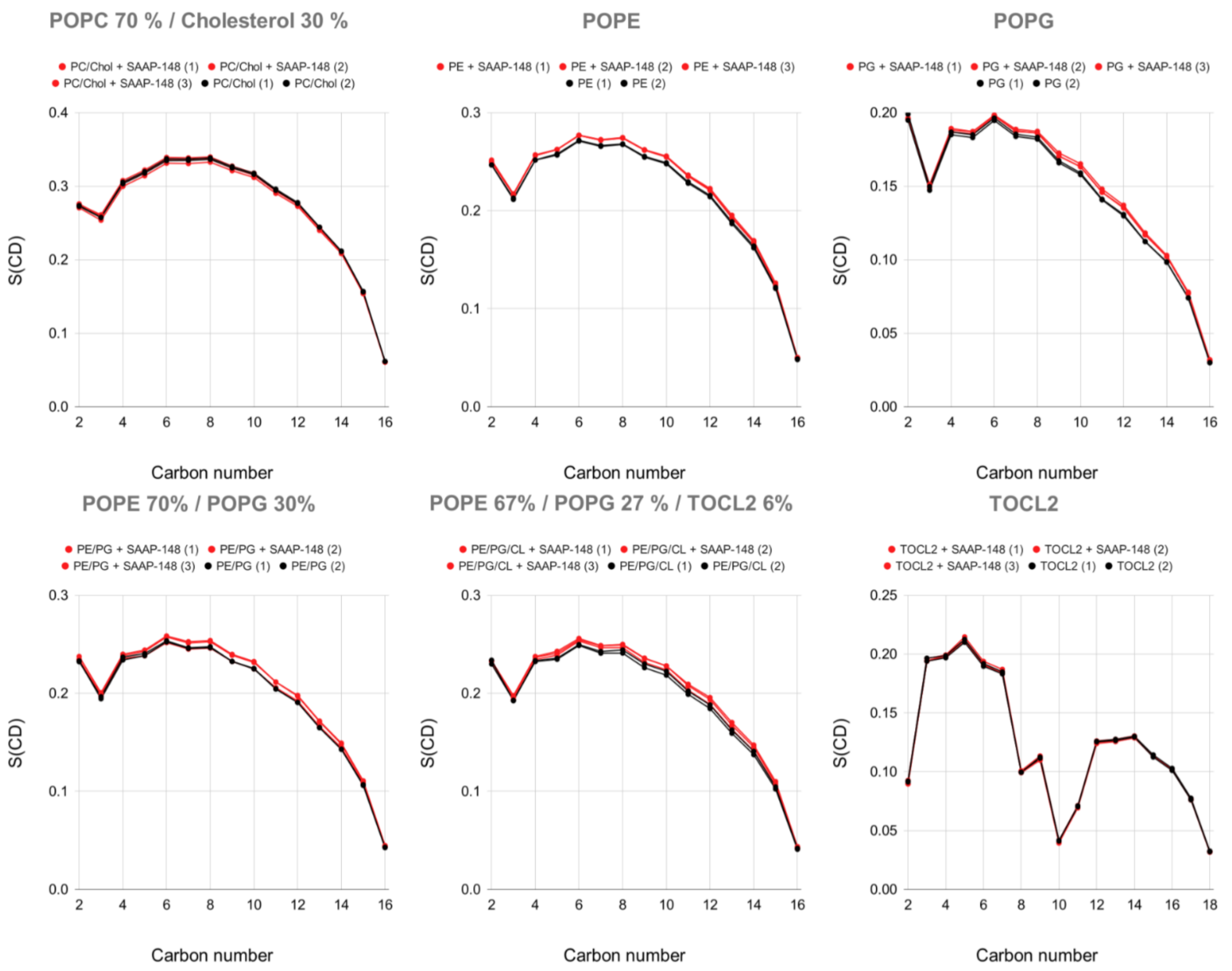

Figure S9. Order parameter for different bilayer models mimicking common bacterial and mammalian lipids with (in red) and without (in black) one molecule of SAAP-148 peptide. Sodium was used as a counterion.

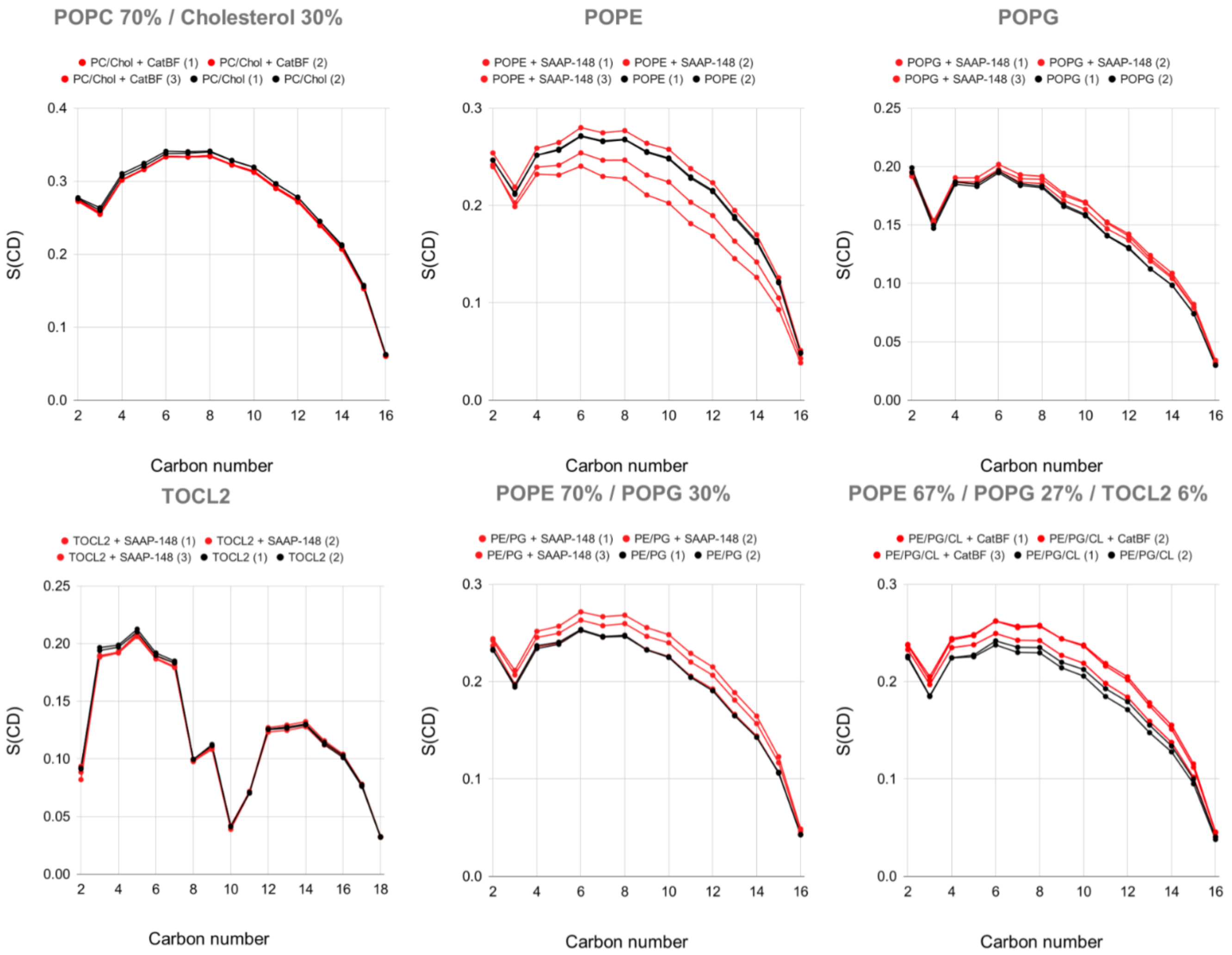

Figure S10. Order parameter for different bilayer models mimicking common bacterial and mammalian lipids with (in red) and without (in black) multiple SAAP-148 peptides. Sodium was used as a counterion.

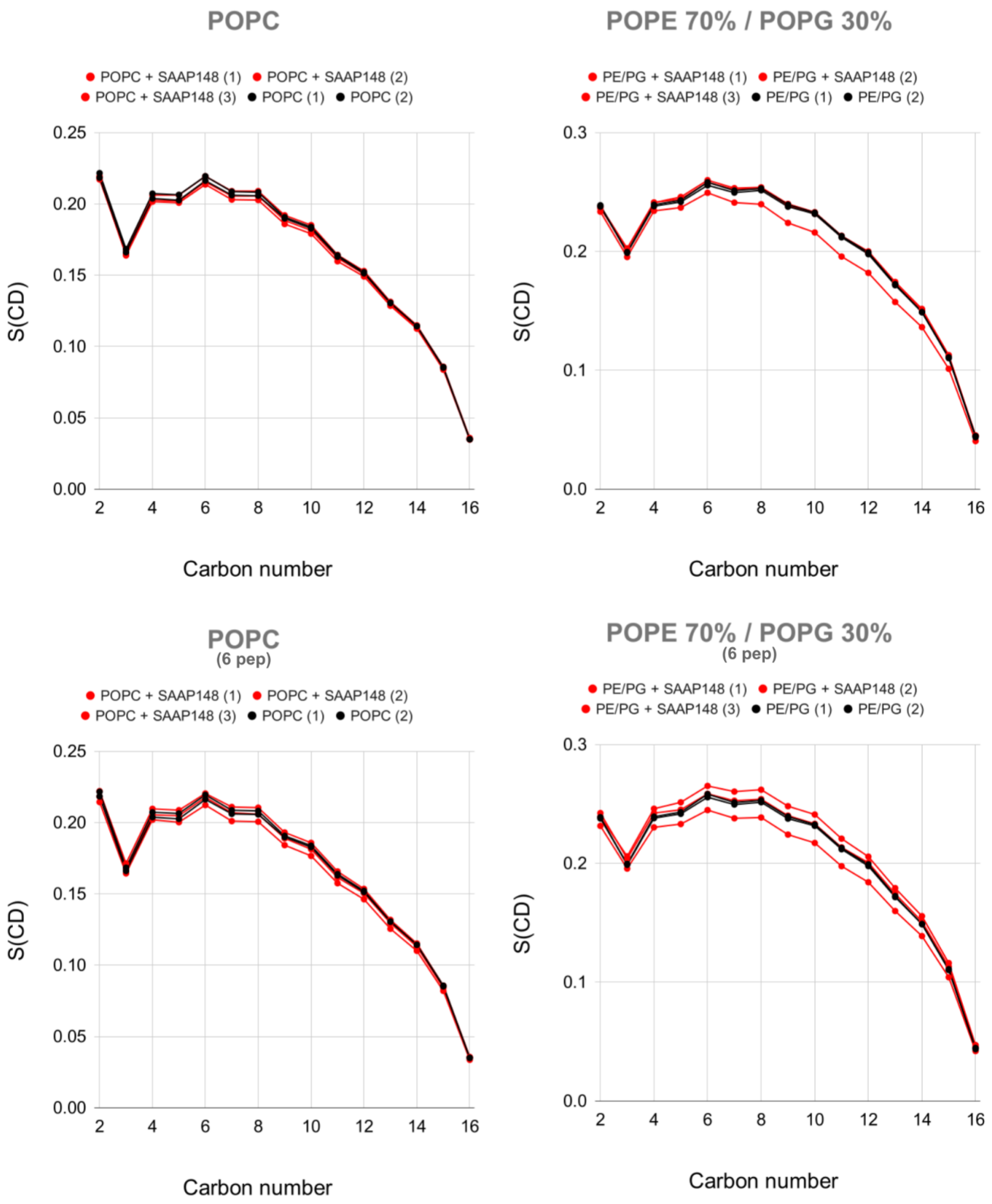

Figure S11. Order parameter for different bilayer models mimicking common bacterial and mammalian lipids with (in red) and without (in black) single (up) or multiple (down) SAAP-148 peptides in presence of calcium.

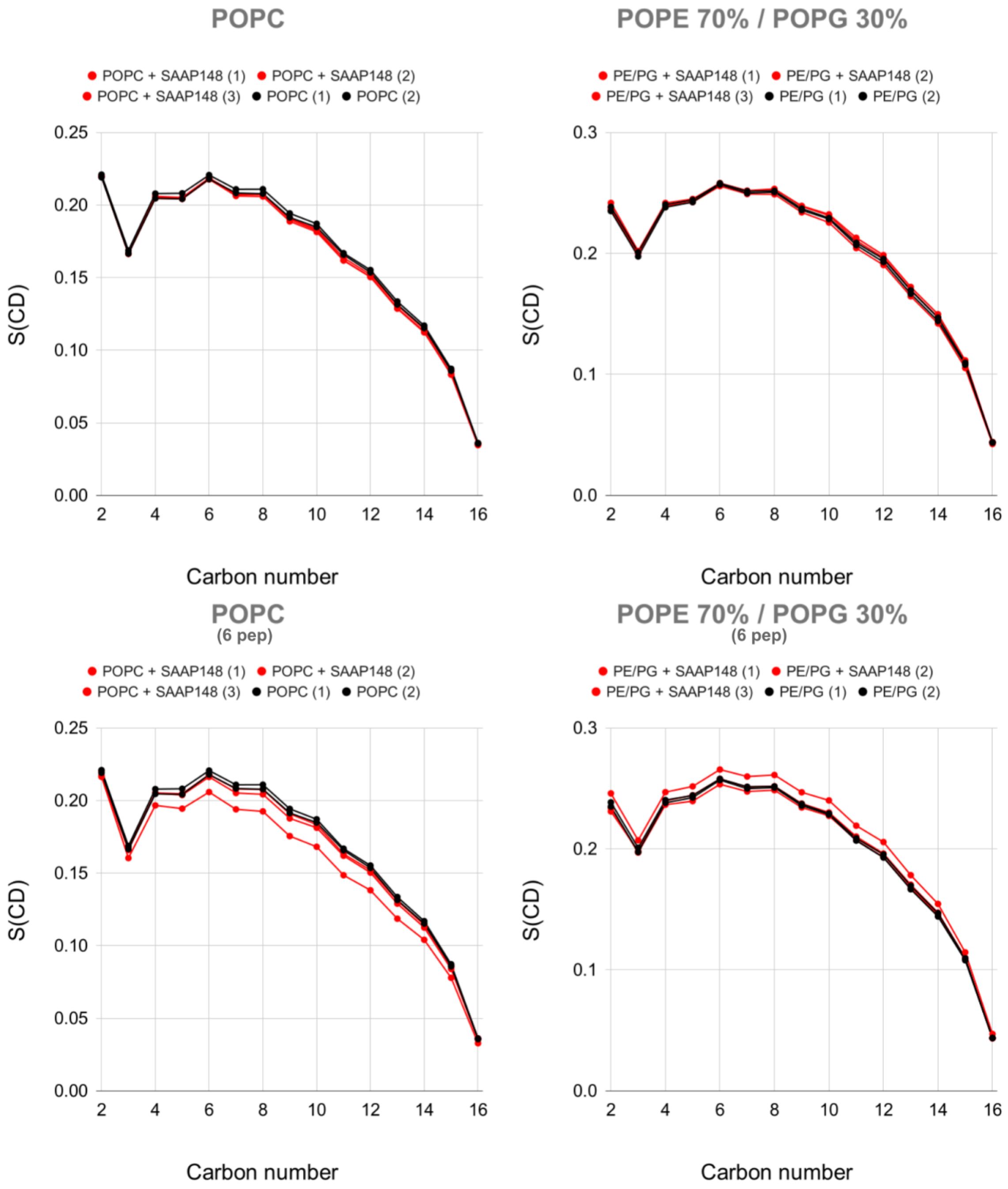

Figure S12. Order parameter for different bilayer models mimicking common bacterial and mammalian lipids with (in red) and without (in black) single (up) or multiple (down) SAAP-148 peptide in presence of magnesium.

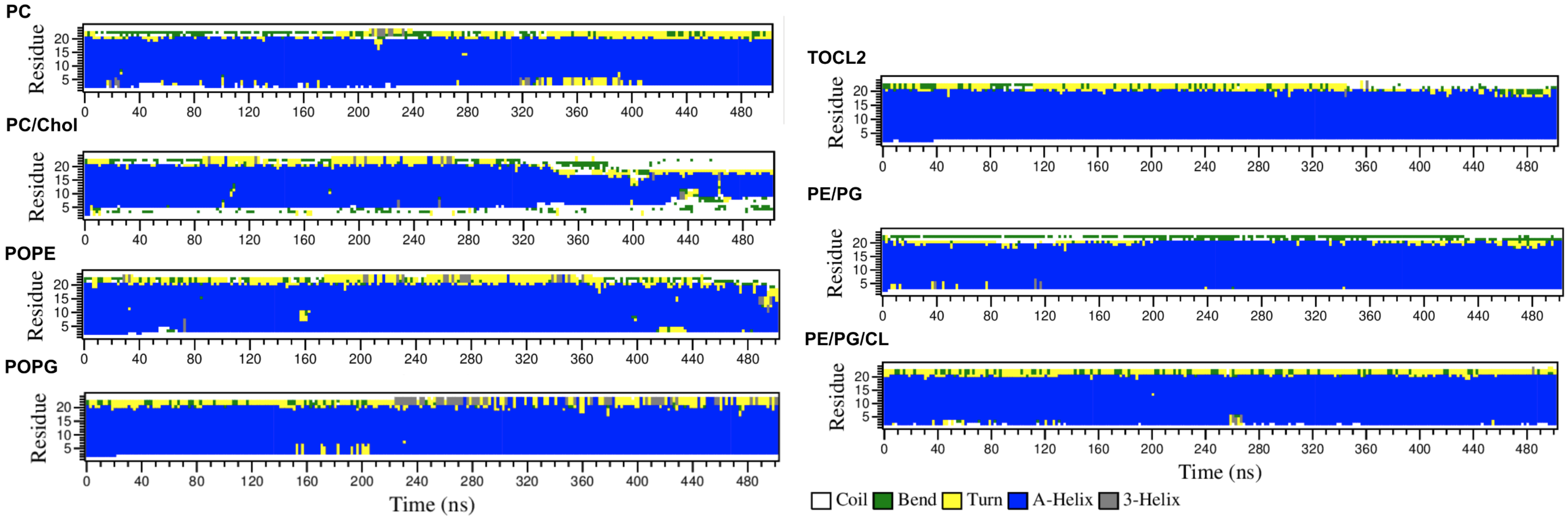

Figure S13. DSSP secondary structures calculated along molecular dynamics (MD) simulations. Sodium was used as a counterion.

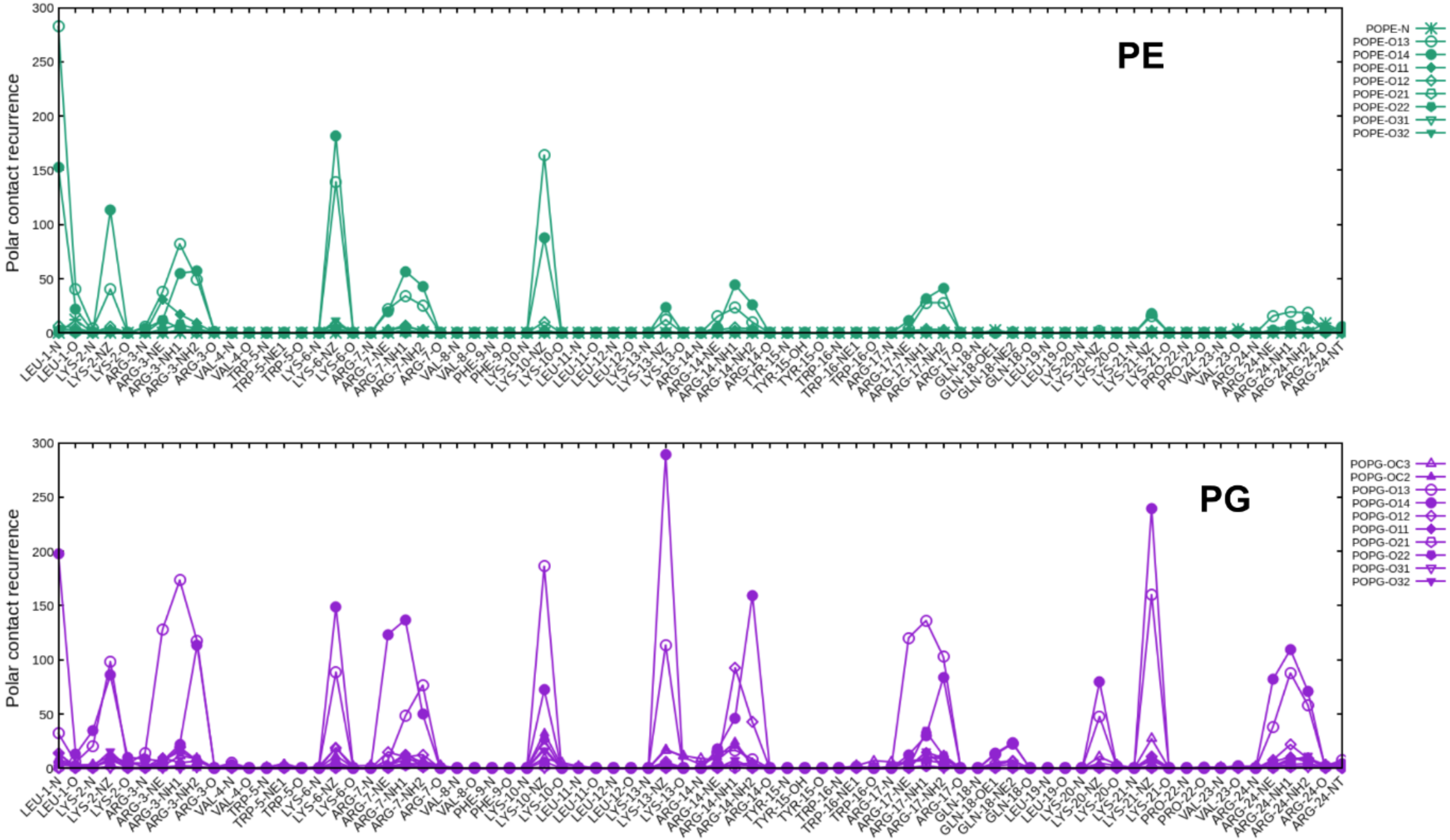

Figure S14. Polar contacts (hydrogen bonds and salt bridges) of SAAP-148 with different lipid bilayers. Sodium was used as a counterion.

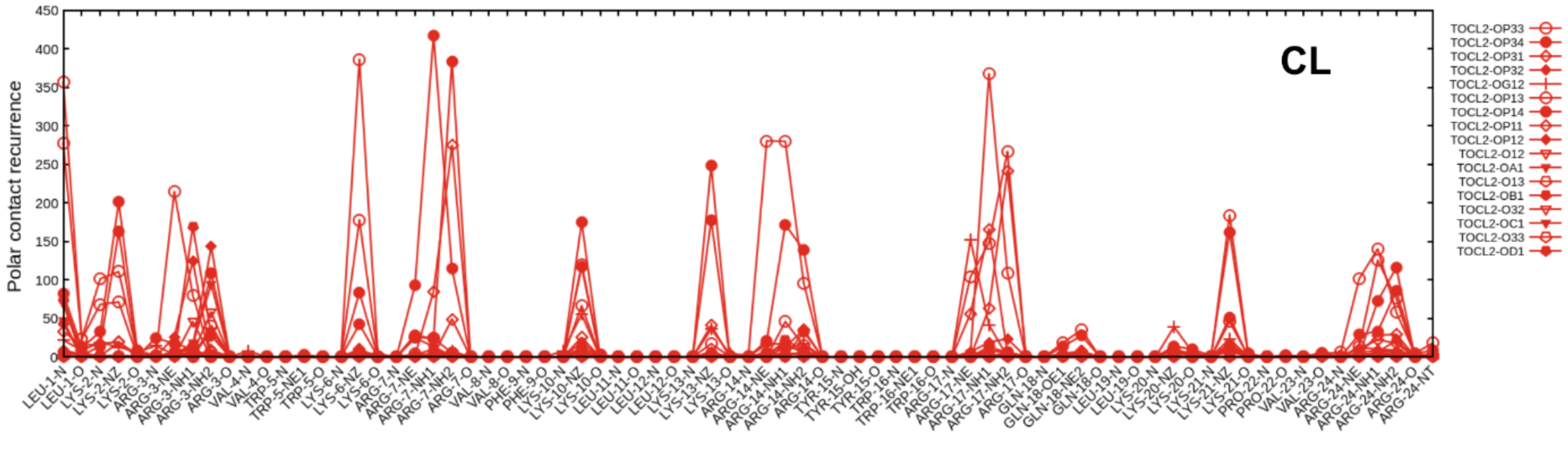

Figure S15. Polar contacts (hydrogen bonds and salt bridges) of SAAP-148 with different lipid bilayers. Sodium was used as a counterion.

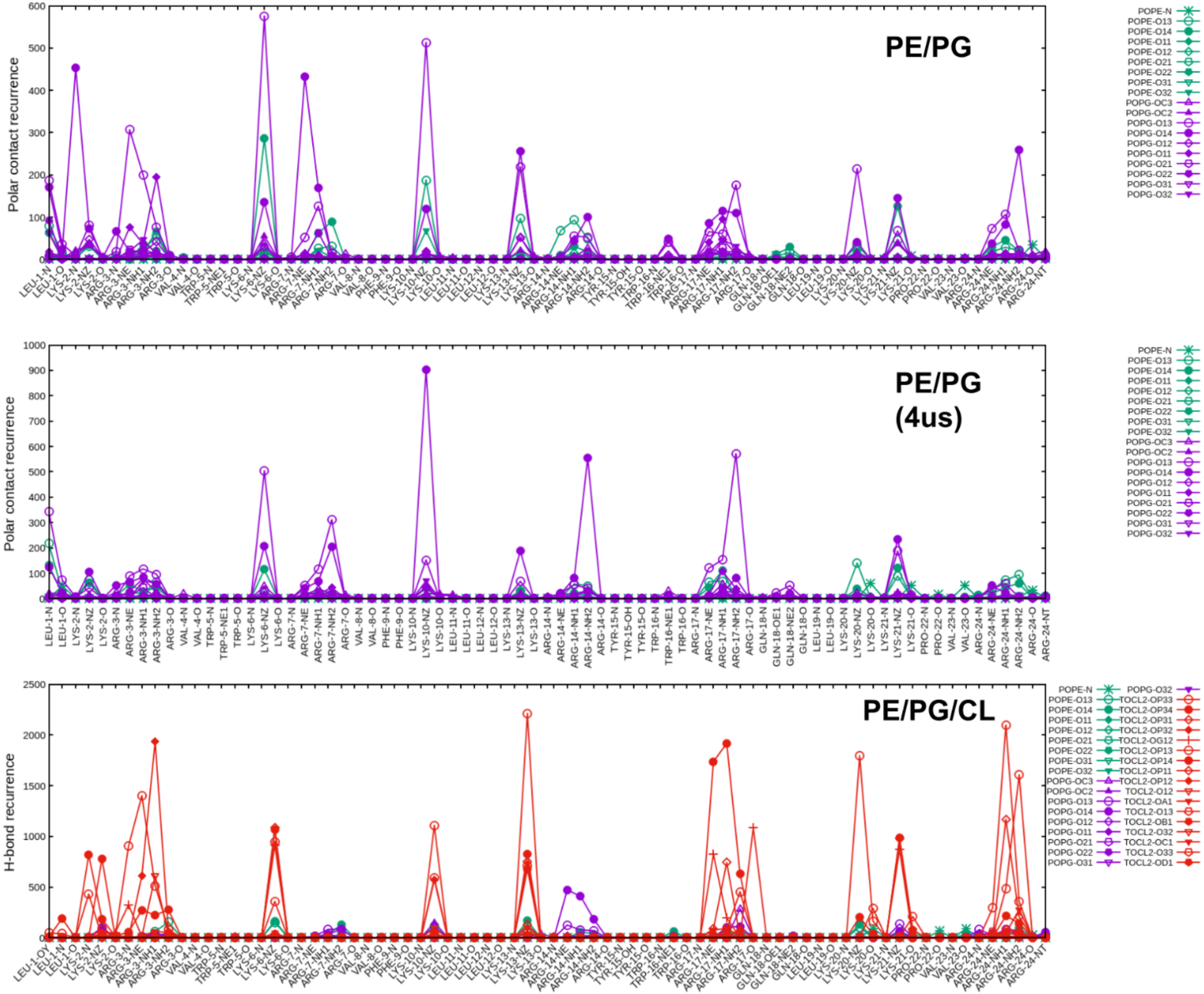

Figure S16. Polar contacts (hydrogen bonds and salt bridges) of SAAP-148 with PE/PG (at 500 ns and 4us) and PE/PG/CL (500 ns) bilayers. Sodium was used as a counterion.

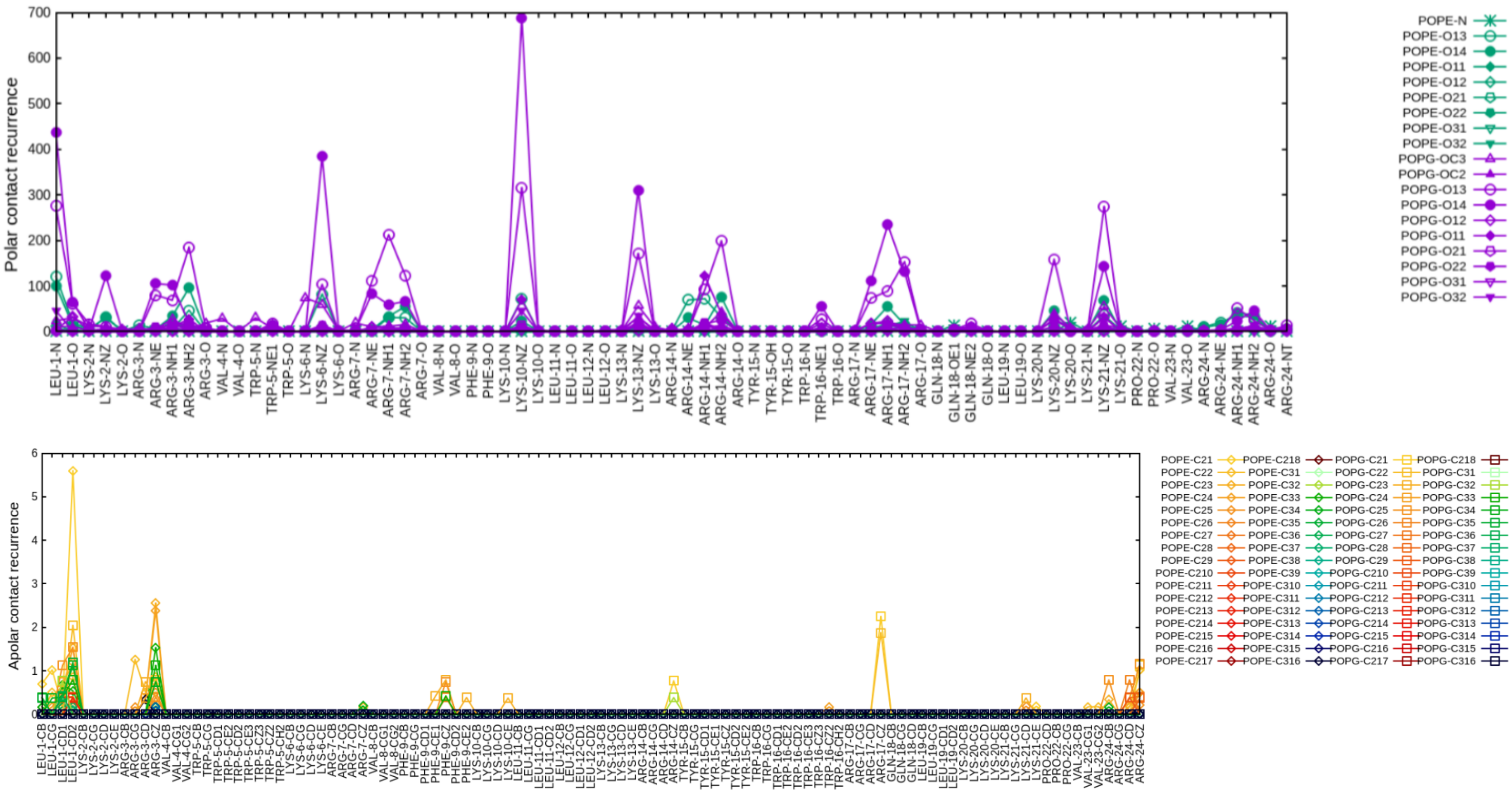

Figure S17. Polar (hydrogen bonds and salt bridges) and apolar (van der Waals interactions) contacts of SAAP-148 with POPE/POPG in presence of magnesium.

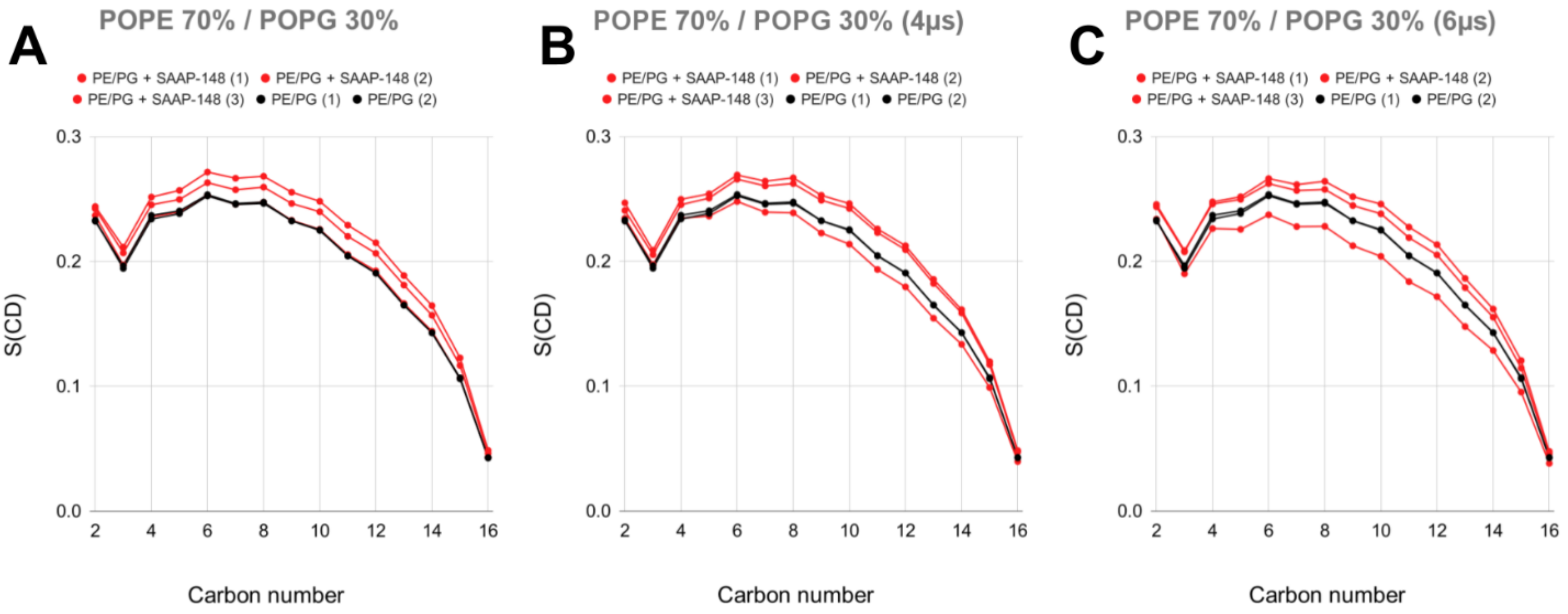

Figure S18. Order parameter for POPE/POPG (70:30) bacterial mimicking bilayers with (in red) and without (in black) SAAP-148 multiple peptides. (A) Slight increase of order parameter at 500 ns, (B) decrease at 4 μs and (C) 6 μs. Sodium was used as a counterion.

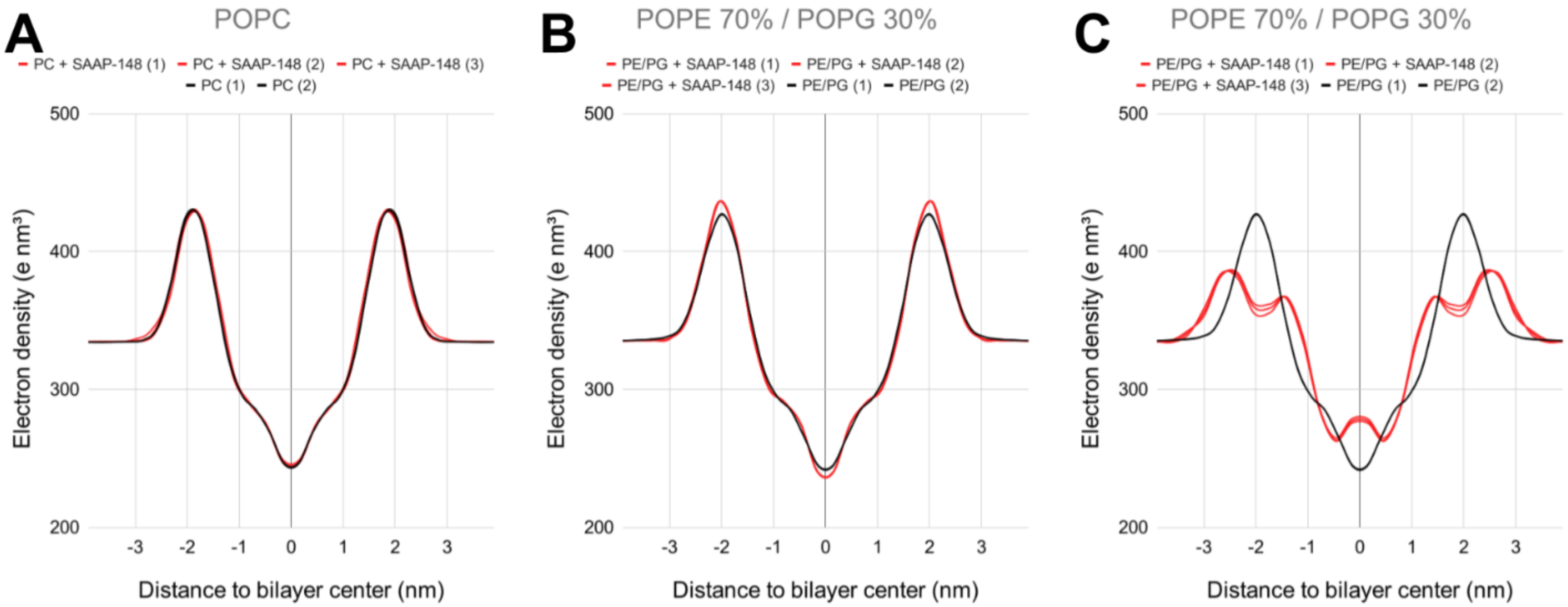

Figure S19. Electron density profiles with (in red) and without (in black) SAAP-148 peptide. (A) POPC model mimicking mammalian membranes, (B) POPE/POPG (70:30) model mimicking bacterial membranes at 500 ns and (C) 4 us. Sodium was used as a counterion.

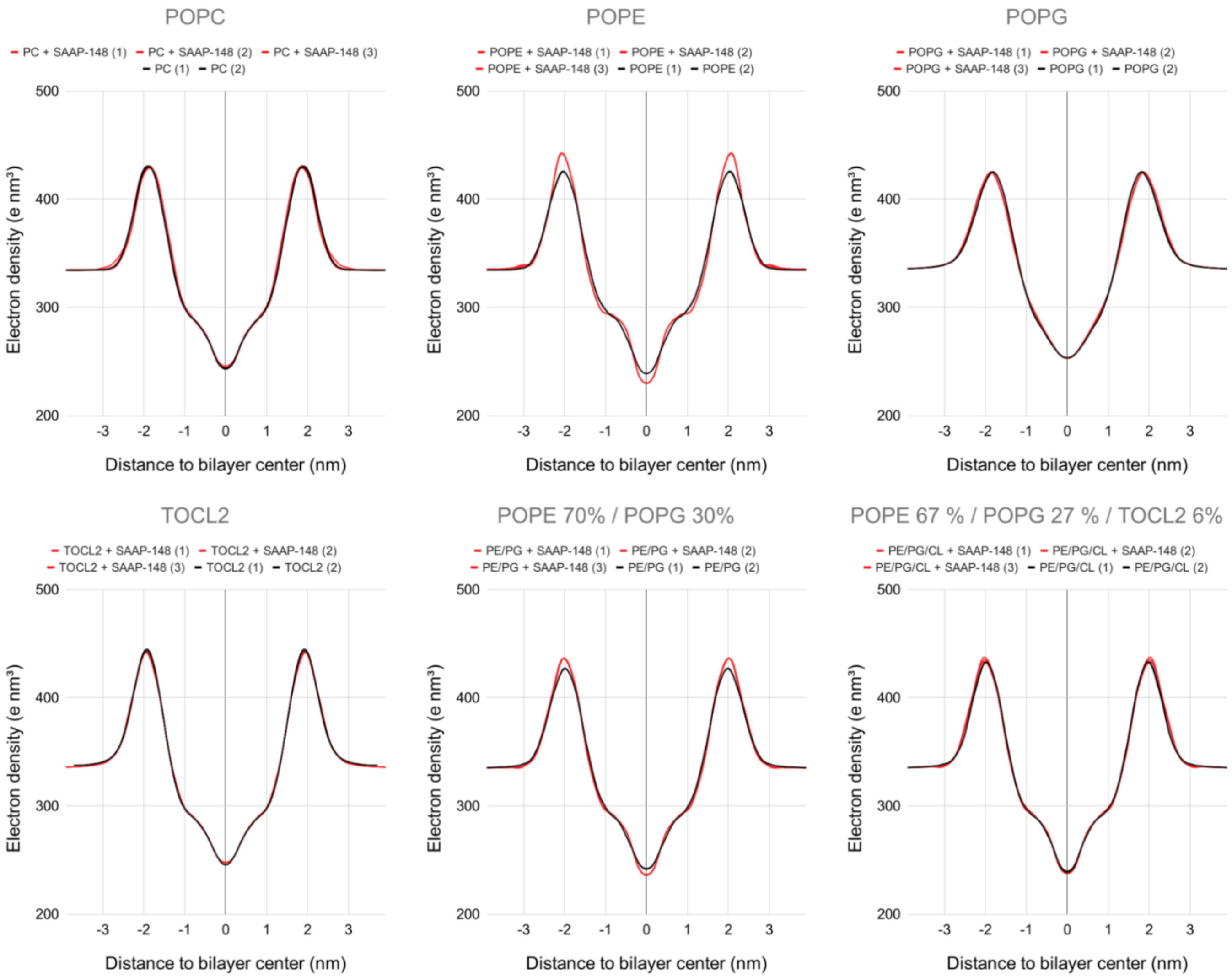

Figure S20. Electron density profiles with (in red) and without (in black) SAAP-148 peptide. For different membrane model bilayers. Sodium was used as a counterion.

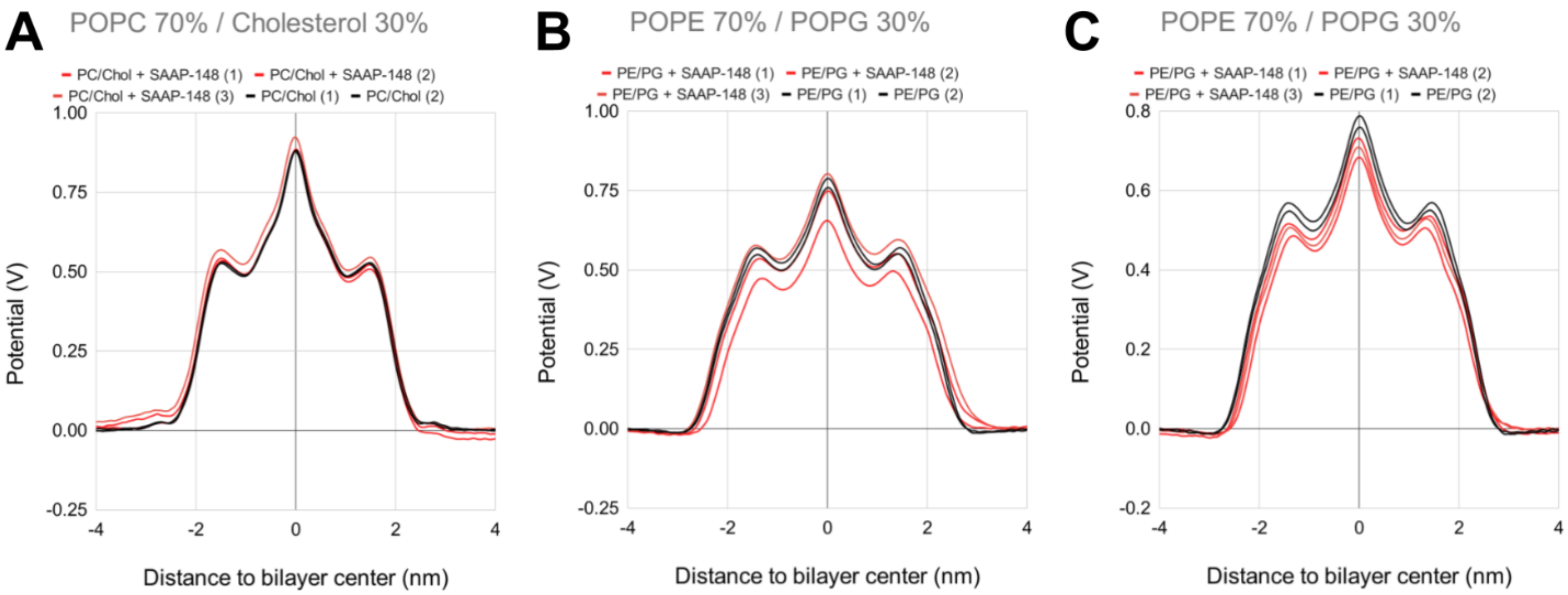

Figure S21. Dipole potential profiles with (in red) and without (in black) SAAP-148 peptide. For different membrane model bilayers: (A) POPC:CHO as mammalian cell model, (B) POPE:POPG up to 500 ns and (C) POPE:POPG up to 4us. Sodium was used as a counterion.

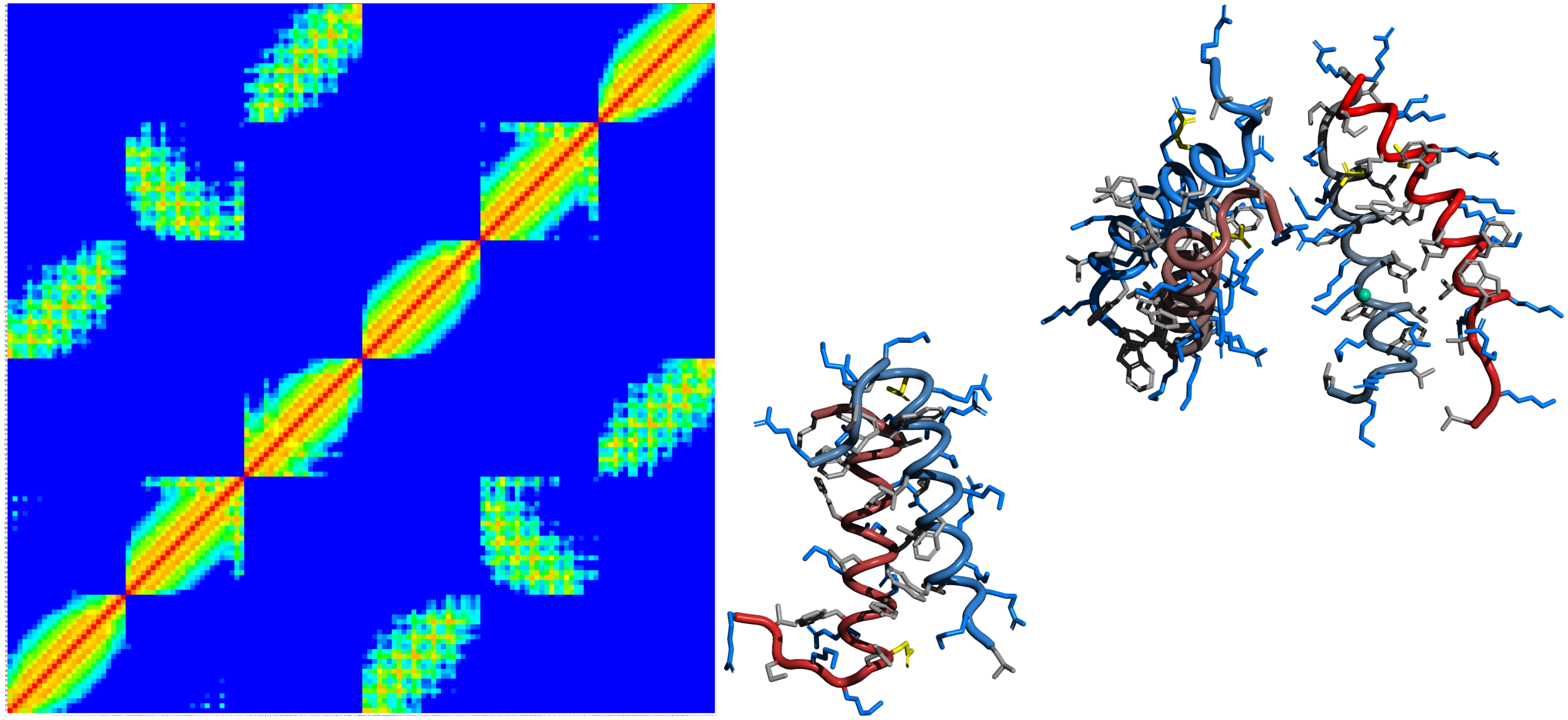

Figure S22. Contact map (left) showing the dimerization effect of SAAP-128 (right). Sodium was used as a counterion.

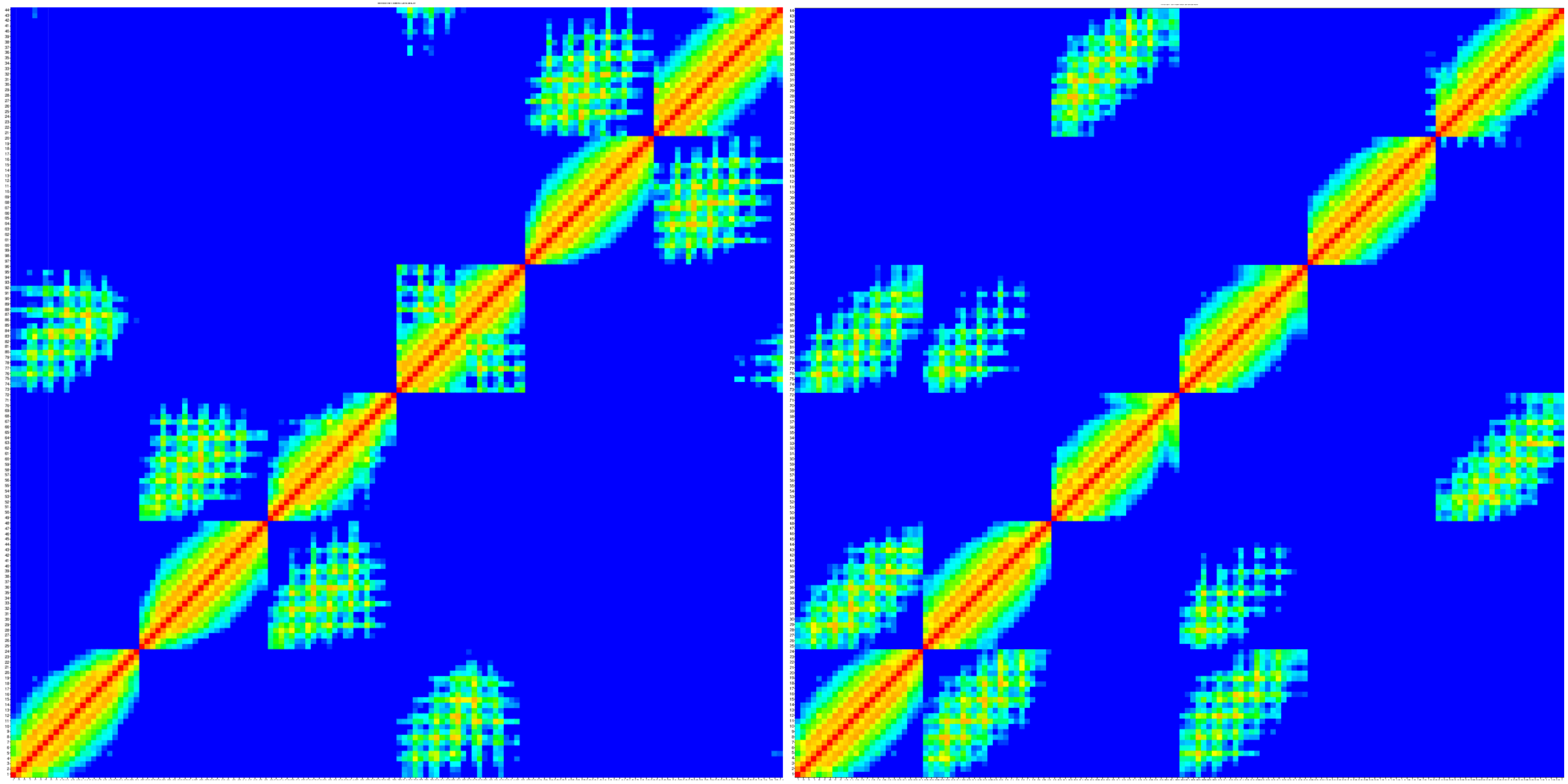

Figure S23. Contact maps showing the aggregation behavior in presence of calcium (left) and magnesium (right).

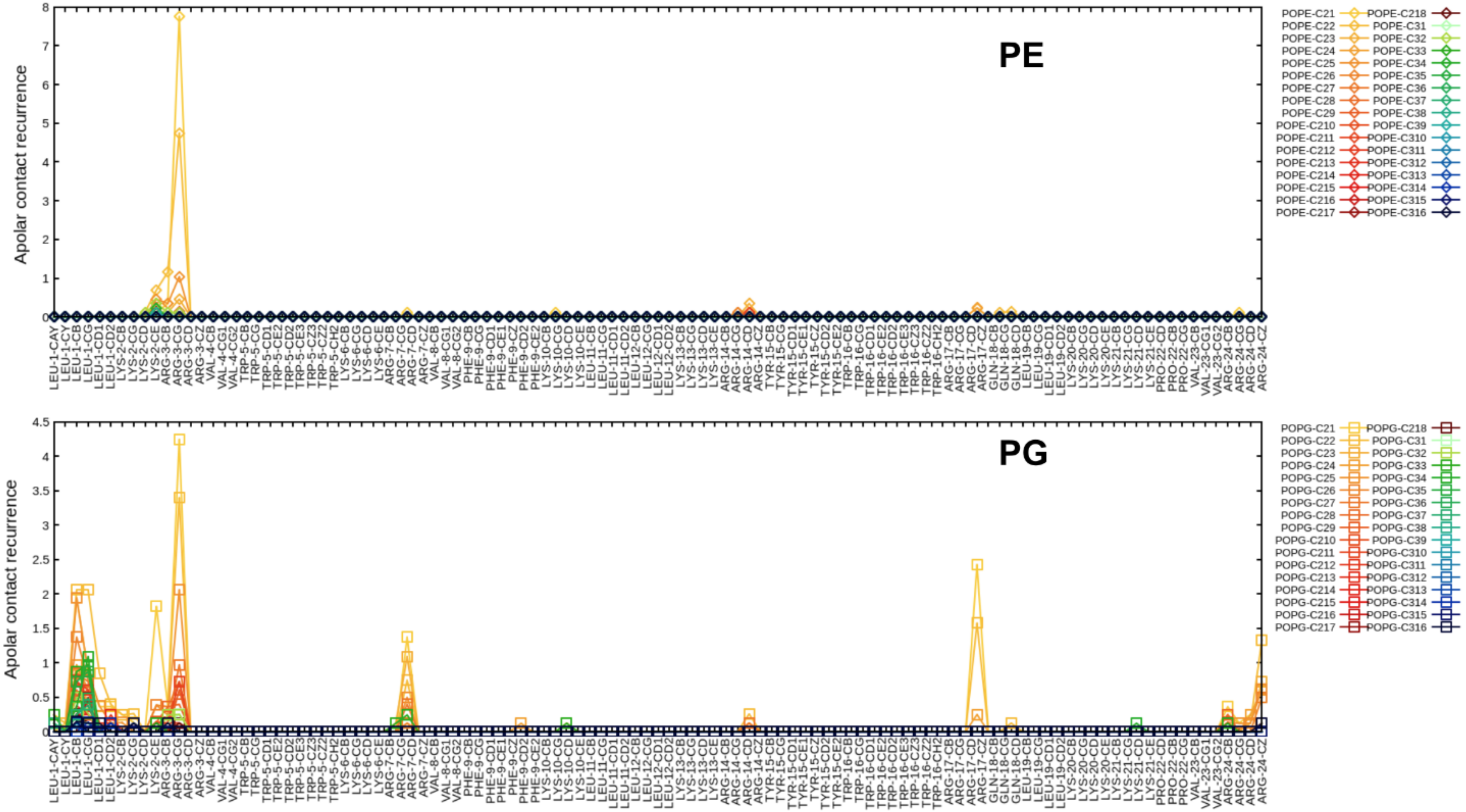

Figure S24. Apolar contacts (van der Waals interactions) of SAAP-148 with different lipid bilayers. Sodium was used as a counterion.

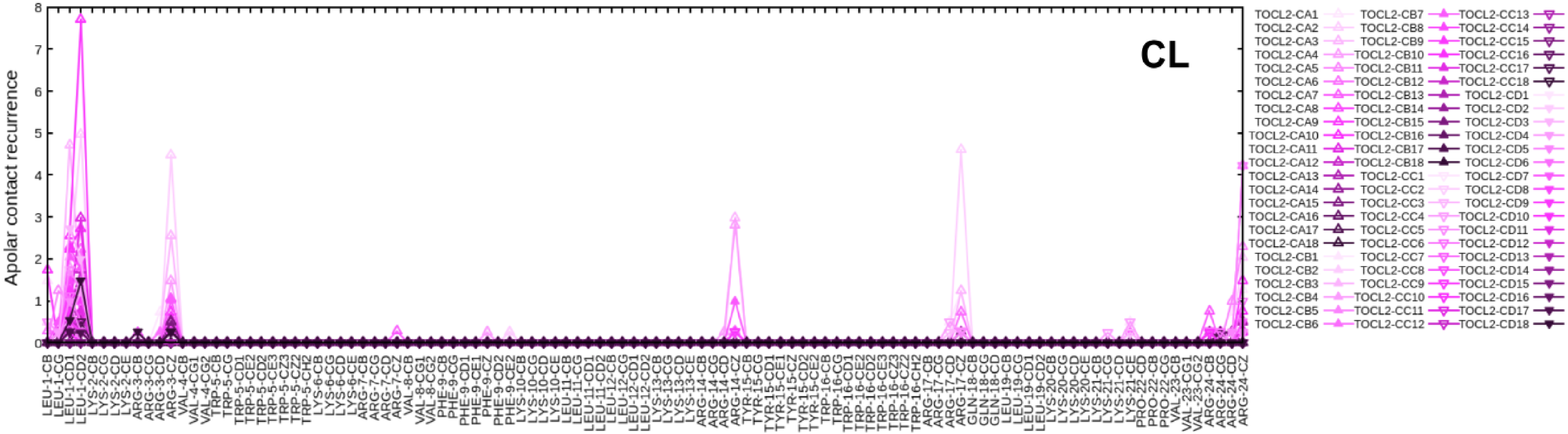

Figure S25. Apolar contacts (van der Waals interactions) of SAAP-148 with different lipid bilayers. Sodium was used as a counterion.

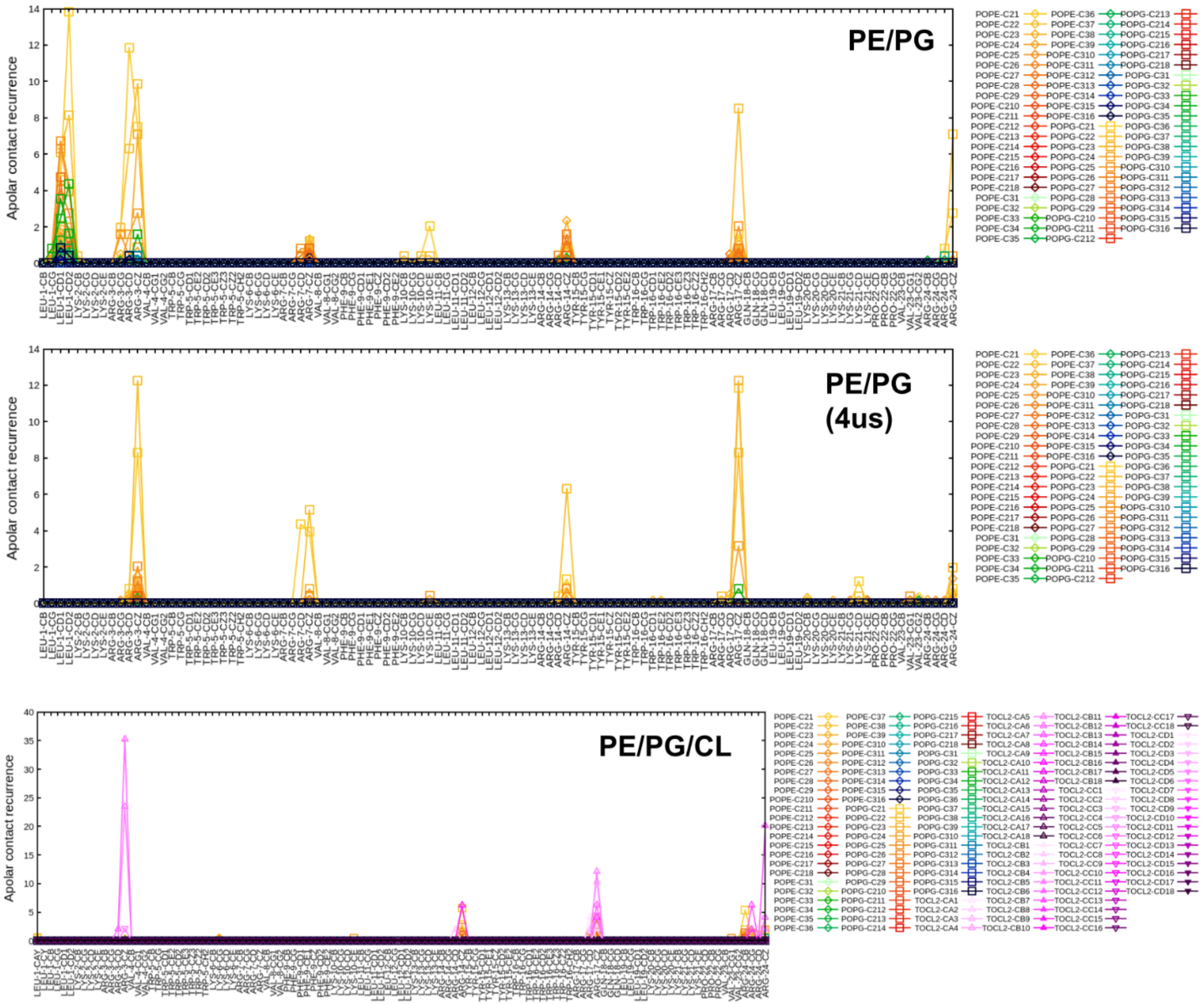

Figure S26. Apolar contacts (van der Waals interactions) of SAAP-148 with PE/PG (at 500 ns and 4us) and PE/PG/CL (500 ns) bilayers. Sodium was used as a counterion.
